# Prolactinoma assessment and management: an international multidisciplinary cross-sectional survey of current practice

**DOI:** 10.1007/s11102-026-01757-8

**Published:** 2026-09-16

**Authors:** Sunita M. C. De Sousa, Hani J. Marcus, Christopher D. Ovenden, Daniel Prevedello, Stephan Petersenn

**Affiliations:** 1 School of Medicine, Adelaide University, Adelaide, South Australia Australia; 2 Endocrine & Metabolic Unit, Royal Adelaide Hospital, Adelaide, South Australia Australia; 3 South Australian Adult Genetics Unit, Royal Adelaide Hospital, Adelaide, South Australia Australia; 4 Department of Neurosurgery, National Hospital for Neurology and Neurosurgery, London, UK; 5 Division of Neurosurgery, UCL Queen Square Institute of Neurology, London, UK; 6 Department of Neurological Surgery, The Ohio State University, Columbus, OH USA; 7 ENDOC Center for Endocrine Tumors, Hamburg, Germany; 8 Department of Medicine, University of Duisburg-Essen, Essen, Germany

**Keywords:** Prolactinoma, Pituitary adenoma, Pituitary neuroendocrine tumour, Dopamine agonists, Surgery, Survey

## Abstract

**Purpose:**

Prolactinomas are the commonest pituitary adenoma encountered in clinical practice, yet many aspects of their assessment and management remain variable. The 2023 Pituitary Society Consensus Statement on the diagnosis and management of prolactin-secreting pituitary adenomas provided important contemporary guidance, but the extent to which current practice aligns with these recommendations remains unclear. We aimed to define international multidisciplinary perspectives on resource availability and clinical decision-making in prolactinoma care.

**Methods:**

We conducted an international cross-sectional survey of endocrinologists and neurosurgeons involved in prolactinoma management. Questions examined access to clinical resources, awareness of emerging issues in prolactinoma care, and areas of concordance or discordance between reported practice and published guidance.

**Results:**

A total of 249 clinicians completed the survey, including 210 endocrinologists and 39 neurosurgeons, from America, Europe, Oceania and Asia. Important resource limitations included inconsistent reporting of Knosp grade and limited availability of transcription factor immunohistochemistry. Only 20% of respondents routinely referred newly diagnosed prolactinoma cases for multidisciplinary team discussion. Most endocrinologists counselled patients regarding dopamine agonist-related risks. Amongst neurosurgeons, reported indications for surgery and views on dopamine agonist-induced tumour fibrosis varied considerably. Although current guidelines support surgery as an equal first-line option in selected microadenomas, cabergoline remained the preferred initial treatment in these scenarios.

**Conclusion:**

International prolactinoma practice is heterogeneous and often discordant with contemporary guidance. Most notably, cabergoline therapy remains the preferred treatment strategy in microprolactinomas, and multidisciplinary discussion of prolactinoma cases is infrequent. This survey dataset serves as a roadmap for future research and clinical service improvement in prolactinoma care globally.

**Supplementary Information:**

The online version contains supplementary material available at https://doi.org/10.1007/s11102-026-01757-8.

## Introduction

Prolactinomas are the most common subtype of pituitary adenoma, comprising approximately 60% of cases in primary care studies [1, 2]. They also account for approximately 20% of aggressive pituitary tumours and 40% of pituitary carcinomas [3]. Prolactinomas feature prominently in the familial pituitary tumour syndromes, being the most common pituitary adenoma subtype associated with pathogenic variants in *MEN1*, *SDHx* and *MAX* [4–7], and the second most common associated with *AIP* pathogenic variants [8]. In addition, dopamine agonists – the traditional gold standard of therapy for prolactinomas – are increasingly recognised as an imperfect therapy with 10–20% of treated patients experiencing dopamine agonist intolerance or resistance [9]. Overall, individuals with prolactinomas form the largest patient group with pituitary disease in clinical practice, with special needs inherent in the tumour type and therapy.

Despite the prominence of prolactinomas in clinical practice, prolactinomas have been significantly understudied relative to other pituitary adenoma subtypes. Between 2011 and 2021, PubMed publications numbered 842 for ‘Cushing’s disease’ and 1,606 for ‘acromegaly,’ compared to only 785 for ‘prolactinoma’. Individuals with prolactinomas, particularly those with dopamine agonist resistance or intolerance (a group comparable in size to all cases of Cushing’s disease), have accordingly been underserved in the pituitary literature. This gap disproportionally affects women, as prolactinomas exhibit the strongest female predominance amongst pituitary adenoma subtypes [9].

The Pituitary Society’s 2023 international guidelines on prolactinoma care addressed and prioritised this underserved, predominantly female population of people with prolactinomas [10]. Building on this, we developed an international multidisciplinary clinician survey to establish international perspectives on clinical decision-making and available resources in prolactinoma care. The survey was designed to address topical issues such as medical versus surgical treatment decision-making, awareness of dopamine agonist toxicities, echocardiography surveillance for valvulopathy, and the possibility of dopamine agonist-induced tumour fibrosis. We aimed to identify potential disparities in access to management resources, levels of awareness regarding new aspects of care, and areas of practice with homogeneity versus heterogeneity. The resultant dataset presented herein is intended to inform future research and clinical initiatives and advocate for the unmet needs of people with prolactinomas.

## Methods

### Survey development

An internationally-applicable clinician survey was developed to address prolactinoma practices amongst both physicians and surgeons. Topic selection, question design and survey dissemination was led by an experienced multidisciplinary team (SD, HM, SP, DP) with significant prolactinoma expertise across endocrinology and neurosurgery, and spanning Australia, the United Kingdom, Europe and the United States. The team drafted an initial topic and question set that was reviewed by an independent pilot group of clinicians representing endocrinology, neurosurgery and reproductive endocrinology. It was determined that the survey content was beyond the scope of reproductive specialists; thus, the final target population was restricted to endocrinologists and neurosurgeons.

The final survey consisted of 38 questions covering clinician demographics, resource access, and biochemical, radiological, histological and therapeutic aspects across a range of microprolactinoma (< 10 mm) and macroprolactinoma (≥ 10 mm) scenarios. Thirteen questions (questions 1–13) were open to both endocrinologists and neurosurgeons. Fifteen questions (questions 14–28) were restricted to endocrinologists, and ten (questions 29 to 38) to neurosurgeons. The content of the survey questions, answer options and subsequent clinician responses are detailed in Supplemental Data.

The study adhered to the published Checklist for Reporting Results of Internet E-Surveys (CHERRIES) framework [11] to ensure transparency and quality of survey design, data collection and result reporting.

### Survey dissemination

The survey was widely distributed through international endocrinology and neurosurgery professional societies, namely: the Endocrine Society of Australia, New Zealand Endocrine Society, Neurosurgical Society of Australasia, European Society of Endocrinology, German Endocrine Society and British Neuroendoscopy Society. The survey was also advertised at the 2025 Pituitary Congress of the Pituitary Society held in San Francisco, US, and the 2025 Australian and New Zealand Pituitary Alliance Annual Meeting held on the Gold Coast, Australia. In addition, the survey was directly sent to a group of over 70 clinicians with pituitary expertise.

The survey was open for a 4-month period from 27 June 2025 to 27 October 2025, and available to access via computer or mobile phone. It was hosted on Microsoft Forms (Washington, United States of America), with linkage to Google Sheets (California, United States of America) to streamline data collection and analysis. The survey was open to fully qualified specialist endocrinologists or neurosurgeons who regularly manage patients with prolactinomas. Survey participants were explicitly advised to answer the survey with regards to their own clinical practice rather than answering on behalf of their centre. To avoid duplicate submissions, participants were instructed to complete the survey only once, and a reminder was given at the end of the survey. No financial reimbursement was offered. Replies were anonymous, but an option was provided for survey participants to record their name to allow acknowledgement in publications.

### Data analysis

For each question, it was determined if a guideline statement was applicable to the topic. Guidelines considered included the 2023 Pituitary Society prolactinoma guidelines [10], the 2017 Pituitary Society Pituitary Tumor Centers of Excellence (PTCOE) guidelines [12], the 2021 European Society of Endocrinology guidelines on pituitary adenoma management in pregnancy [13], and the 2019 joint statement on valvulopathy screening during dopamine agonist therapy by the British Society of Echocardiography, the British Heart Valve Society and the Society for Endocrinology [14]. Additional publications were selected for comparison with certain responses where relevant.

Collective responses were classified as reaching consensus if ≥ 60% of survey participants gave the same response; if the most frequent answer was given by < 60% of participants, this was classified as variability in practice. The threshold of 60% to define consensus was employed as this has been considered reflective of moderate agreement in consensus studies [15]. Collective responses were also assessed as to whether they were concordant with the aforementioned guidelines.

Statistical analysis involved Chi-square tests to investigate associations between factors such as geographical location or specialty of participants and their responses.

## Results

### Study population

A total of 249 participants completed the survey, consisting of 133 (53%) participants from Europe, 67 (27%) from Oceania, 29 (12%) from North America, 17 (7%) from Asia and 3 (1%) from South America. By specialty, 210 (84%) participants were endocrinologists and 39 (16%) were neurosurgeons. Collated responses to all survey questions, including additional demographic and prolactinoma case load details, are outlined in Supplemental Data.

### Resource availability

Although 83% of participants stated that they can directly refer patients with prolactinomas for multidisciplinary team (MDT) discussion, only 20% stated that they refer most or all patients with a newly diagnosed prolactinoma for MDT discussion regarding first-line therapy. Prolactinoma imaging generally appeared to be of high quality with most participants stating that their local radiology centres usually included: gadolinium administration (96%), reporting of the maximal diameter (93%), fine cut imaging through the pituitary (92%), dynamic sequence imaging (70%) and review by a neuroradiologist (67%). Only 39% of participants reported that Knosp grade was usually included. Prolactinoma staining studies were reported to be usually performed for pituitary hormones in 96%, Ki-67 in 88% and transcription factors in 64%. Participant access to radiotherapy varied by modality, from proton beam therapy in 20% to conventional fractionated radiotherapy in 86%. When germline genetic testing is indicated, 54% of participants reported being able to refer patients to a local clinical genetic service, 36% arranged genetic testing independently, 6% had no access to genetic testing and 4% were uncertain regarding access. All participants reported local access to cabergoline, whereas only 66% and 23% of participants reported routine availability of bromocriptine and quinagolide, respectively. A typical surgery wait time of > 4 weeks for functioning microadenomas was reported by 59% of neurosurgeon participants.

### Clinician awareness

Prolactinoma caseload exposure amongst endocrinologists was variable, with typical caseloads of < 2 per month reported by 31/210 (15%), 2–4 per month by 89/210 (42%), and > 6 per month by 89/210 (42%) of endocrinologists. Most endocrinologists (> 50%) reported advising patients either routinely or sometimes of the potential dopamine agonist-induced consequences of gastrointestinal and vasodilatory side effects, mood disturbance, impulse control disorders, cardiac valvulopathy, CSF rhinorrhoea and return of fertility. Most endocrinologists also reported either routinely or sometimes discussing the option of switching to surgical management in the event of dopamine agonist-induced nausea, anorexia, postural lightheadedness, depression, impulse control disorders or persistent or progressive prolactinomas after 2 years of maximally tolerated dopamine agonist therapy. However, 63% of endocrinologists explicitly stated that they would not consider surgical management because of dopamine agonist-induced fatigue.

Amongst neurosurgeon participants, 82% had completed pituitary subspecialty training. The median number of pituitary operations performed yearly was 60 (range 5-200). The most frequently observed indication for prolactinoma surgery was variable: 49% dopamine agonist intolerance, 23% patient preference, 18% dopamine agonist resistance and 10% ‘other’. Neurosurgeon reported rates of hampered prolactinoma resection due to apparent bromocriptine-associated tumour fibrosis were: 11% almost always or frequent, 23% occasional, 18% almost never and 49% uncertain. Rates of hampered prolactinoma resection due to apparent cabergoline-associated tumour fibrosis were: 18% almost always or frequent, 41% occasional, 26% almost never and 15% uncertain.

### Assessment and management practices

**Fig. 1:**
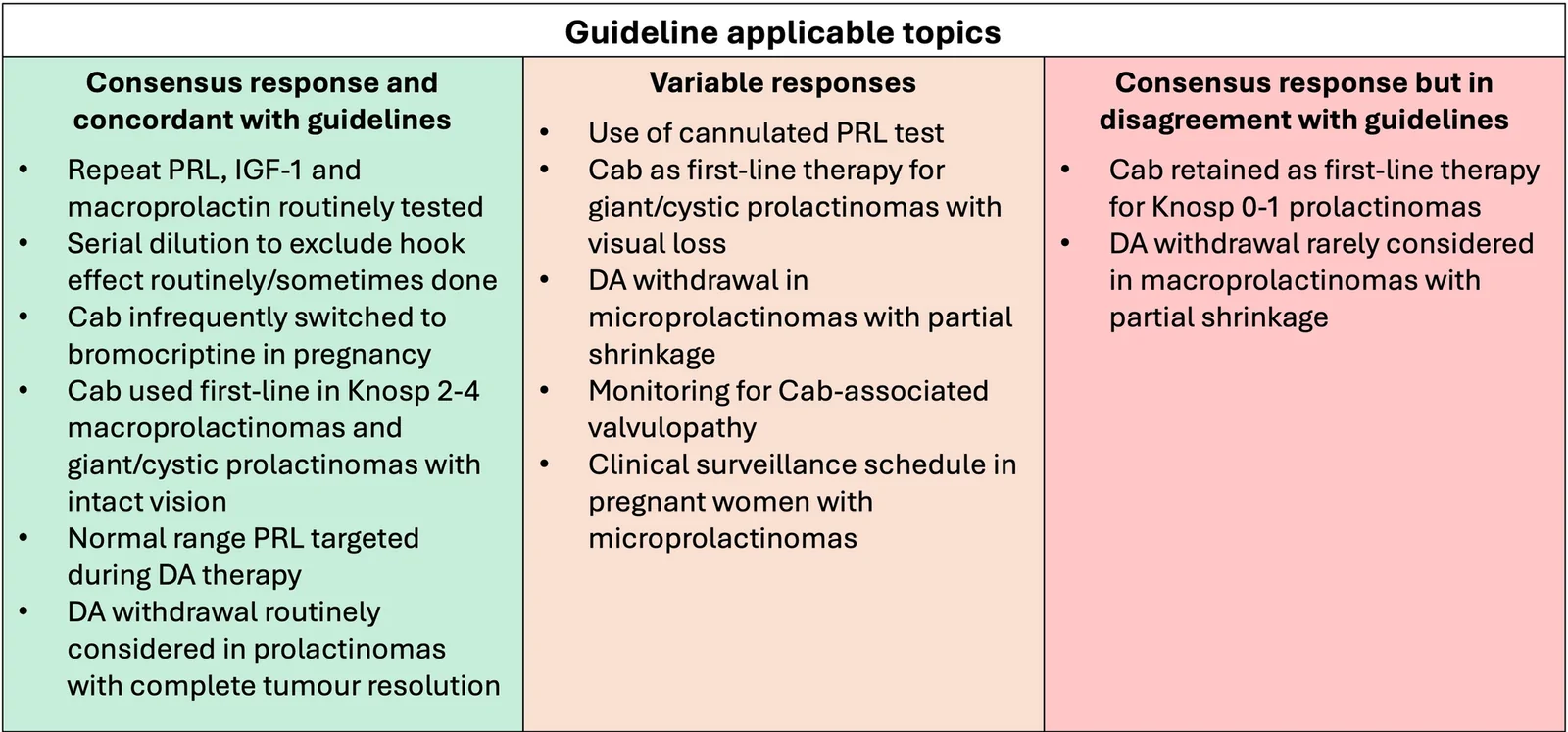
Summary of responses to questions with applicable guidelines. The first box indicates areas of consensus that are consistent with guidelines. The second and third boxes indicate areas that may be considered for promotion of previous guidelines, consideration of guideline revisions and/or future research directions given the discord between reported practice and existing guidelines. Abbreviations: Cab, cabergoline; DA, dopamine agonist; PRL, prolactin

**Fig. 2:**
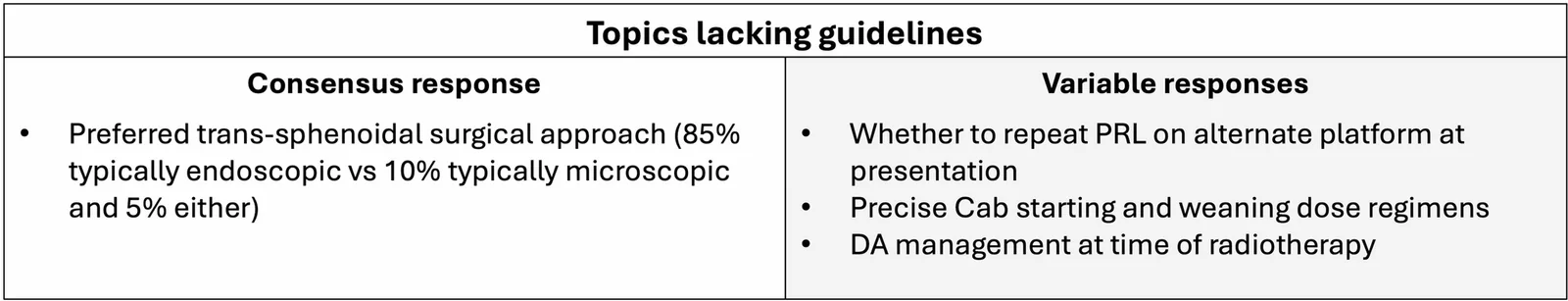
Summary of responses to questions without applicable guidelines. The first box indicates areas for consideration in future guideline development given the consensus opinion and lack of coverage in past guidelines. The second box indicates potential future research directions given the lack of both consensus opinion and applicable guidelines. Abbreviations: Cab, cabergoline; DA, dopamine agonist; PRL, prolactin

Figure 1 maps survey responses to topics with applicable guidelines, according to whether consensus was reached and whether the responses were concordant with guidelines. Figure 2 maps survey responses to topics lacking applicable guidelines, according to whether consensus was reached or not.

In the majority of cases, a guideline existed to inform practice; in these situations, most responses reached consensus and were concordant with guideline recommendations. In agreement with the 2023 Pituitary Society guidelines [10], this included: routine performance at initial presentation of repeat serum prolactin to confirm sustained hyperprolactinaemia (90%), IGF-1 measurement to screen for growth hormone hypersecretion (85%) and PEG precipitation to screen for macroprolactinaemia (62%); infrequent switching from cabergoline to bromocriptine in the setting of pregnancy (only 2% in microprolactinomas, 18% in macroprolactinomas); primary therapy with cabergoline for macroprolactinomas with Knosp grade 2–4 cavernous sinus invasion and giant or cystic prolactinomas with intact visual fields (> 78% of participants across these indications); targeting a normal range serum prolactin during dopamine agonist therapy; and considering dopamine agonist withdrawal in prolactinomas with normalised prolactin and complete tumour resolution after 2 years of dopamine agonist therapy. In addition, 75% of participants reported that they routinely or sometimes order serial dilution to exclude the high-dose hook effect as a cause of a falsely low prolactin in massive hyperprolactinaemia due to a large prolactinoma. This practice is in accordance with the 2023 Pituitary Society guidelines [10] but contrary to a French multi-platform study showing that the hook effect is seldom seen with modern assays [16].

In two areas, responses reached consensus but were in disagreement with current guidelines. Firstly, > 70% of participants reported that they would initially treat various microprolactinoma scenarios and macroprolactinomas with Knosp grade 1 cavernous sinus invasion (question 12a-d) with cabergoline rather than presenting medical and surgical management as equal options in these scenarios as recommended in the 2023 Pituitary Society prolactinoma guidelines [10]. Specifically, 76% of participants advised that they would initially treat a Knosp 1 prolactinoma with cabergoline. Treatment choice in this scenario was not associated with practice location (*p* = 0.10) or number of cases treated monthly (*p* = 0.23). However, treatment choice was associated with specialty (*p* = 0.02) as neurosurgeons were significantly more likely than endocrinologists to recommend surgery (13% vs. 3%) or equal options of surgery or medical therapy (26% vs. 16%) rather than cabergoline therapy (59% vs. 79%). Secondly, 88% of respondents reported they would not consider dopamine agonist withdrawal in macroprolactinomas with a normalised prolactin but only partial tumour shrinkage after 2 years of dopamine agonist therapy, though consideration of treatment withdrawal has been recommended in this setting [10].

In some cases, a guideline existed to inform practice but there was variability in survey responses. In regard to the assessment of hyperprolactinaemia, 55% of participants explicitly stated that they would not use a cannulated prolactin test (falling slightly below the consensus threshold of 60%), contrasting against the Pituitary Society recommendation to consider this test if stress hyperprolactinaemia is suspected [10]. Regarding prolactinoma management, only 53% of respondents said they would use cabergoline as first-line therapy for a giant (≥ 40 mm) prolactinoma with visual loss despite the Pituitary Society guidelines including such cases for consideration of cabergoline [10]. Similarly, only 38% of respondents said they would pursue cabergoline in the setting of cystic macroprolactinomas with visual loss despite the Pituitary Society advising that dopamine agonist therapy should be considered as a viable option in this setting, particularly in patients without urgent need of optic chiasm decompression [10]. There was also variability in whether respondents would withdraw therapy after 2 years of therapy in a microprolactinoma with a normalised prolactin but only partial tumour shrinkage, with 56% saying they would not withdraw therapy, 39% saying they would and 5% ‘unsure’. There was substantial variability in the monitoring of cabergoline-associated valvulopathy both at baseline and follow up. Noting that the corresponding guidelines are UK-based [14], we considered whether there was an association between participant location and these answers, but there were insufficient numbers of UK respondents to perform this analysis. There was also variability in the clinical surveillance schedule in pregnant women with microprolactinomas, potentially reflecting discordance in existing guidelines that either recommend [10] or do not recommend [13] routine endocrinological follow-up during pregnancy.

There were few questions for which an applicable guideline did not exist. Amongst these, there was variability in whether clinicians would repeat prolactin measurement on a different platform at the time of diagnosis (10% yes, 54% no, 8% sometimes, 1% unsure), typical cabergoline starting dose regimens (44% 0.25 mg twice weekly, 24% 0.5 mg weekly, 20% 0.25 mg weekly, 7% 0.5 mg twice weekly, 4% other) and weaning dose regimens (21% no wean before cessation, 14% wean to 0.5 mg weekly then stop, 52% wean to 0.25 mg weekly then stop, 13% wean to 0.25 mg monthly then stop), and whether to stop cabergoline before radiotherapy (25% yes, 51% no, 24% not involved in such cases). Though no guideline exists to support endoscopic over microscopic resection of prolactinomas, a significant majority of participants (85%) stated that they solely resected these tumours endoscopically.

## Discussion

This is the first survey study to evaluate multidisciplinary practices in prolactinoma care. With 249 participants across endocrinology and neurosurgery and representation from Europe, Oceania, America and Asia, this large and internationally representative dataset demonstrates the extent of clinical resource availability, clinician awareness around new aspects of care and the degree of application of guidelines on this topic. The two principal findings of this dataset are related: firstly, that patients with prolactinomas are infrequently reviewed in MDT meetings at the time of initial presentation and treatment decision-making; and secondly, that surgery is infrequently considered as a first-line therapy for prolactinomas, even in Knosp grade 0 and 1 microadenomas.

The major resource limitation identified in this study was the infrequent (20%) referral of patients with prolactinomas to MDT meetings. This is despite macroprolactinomas in men inherently representing an aggressive pituitary tumour subtype expected to require multimodal therapies [17], and recent guidance from the Pituitary Society that microprolactinomas should be considered for surgical resection following discussions in a multidisciplinary setting [10]. Given that the suitability of upfront surgery for microprolactinomas rests on access to expert multidisciplinary care and high-volume neurosurgeons with excellent remission rates, we believe there is a strong imperative to ensure equitable access to MDT meetings and clinics for patients with prolactinomas, so that surgical and medical options can be weighed appropriately. We acknowledge, however, that any recommendation for upfront surgical resection in Knosp grade 0–1 microprolactinomas must be weighed against legitimate clinical concerns, including the risk of postoperative new-onset hypopituitarism, particularly in premenopausal women in whom this may adversely affect fertility, and that such considerations may reasonably temper enthusiasm for a surgery-first approach even where expert multidisciplinary resources are available. Our findings are therefore better read as an argument for equitable access to balanced multidisciplinary discussion, rather than as advocacy for surgery as a default first-line therapy. Expert MDT involvement is particularly valuable in its opportunity for careful radiological review, noting the frequent (55%) omission of Knosp grade reporting in prolactinoma imaging reported by survey participants despite a Knosp grade of 0–1 being a key requirement for primary surgical management of prolactinomas [10], with higher Knosp grades associated with reduced utility from surgical intervention [18]. This is further compounded by the recent finding of suboptimal accuracy of Knosp grade reporting in real-world practice compared to dedicated neuroradiologist review [19], which may represent time pressures on reporting radiologists and/or reduced awareness of the impact of Knosp grading on pituitary adenoma management. Another consideration in the uptake of upfront surgery for prolactinomas is that most neurosurgeons reported a surgical wait time of > 4 weeks for functioning microadenomas. Noting that this group includes patients with Cushing’s disease and acromegaly who are expected to be in greater need for early surgery than patients with microprolactinomas, this means that surgery for prolactinomas is often delayed, during which time cabergoline therapy may be attempted prior to switching to surgery if/when required in accordance with the proposed low surgical threshold model of prolactinoma care [9].

Other notable resource limitations included access to tumour transcription factor immunohistochemistry amongst only 64% of participants despite this being a cardinal component of the 2022 WHO classification of pituitary tumours [20], and heterogenous access to radiotherapy modalities and genetic testing for familial pituitary tumours influencing how recommendations in these areas of care can be feasibly enacted.

Clinician awareness appeared generally good in the survey dataset. Most neurosurgeons reported pituitary subspecialty training, and most endocrinologists reported counselling patients regarding the key potential consequences of dopamine agonist therapy. The high rate of counselling regarding the risk of dopamine agonist-induced impulse control disorders reflects recent studies in this area [21–23], and contrasts against older prolactinoma guidelines when this risk was not recognised [24, 25]. However, there appeared to be underappreciation of the degree of dopamine agonist-induced fatigue that some patients may experience which, anecdotally, may be intolerable or interfere with daily function to the extent that an attempt at surgical management may be preferred. As in the general pituitary literature, prolactinomas are also the most understudied functioning pituitary adenoma subtype in patient reported outcome measure studies [26]; such studies should help define the burden of dopamine agonist-induced fatigue and other side effects. Further studies are also required to explore the potential for cabergoline-induced tumour fibrosis, with 59% of surgeons reporting that they felt tumour resection was hampered by cabergoline almost always, frequently or occasionally despite evidence to date arguing against prolactinoma fibrosis in the cabergoline setting [27, 28].

Notably, 75% of participants reported routinely or sometimes ordering serial dilution to exclude the high-dose hook effect in massive hyperprolactinaemia, in keeping with the 2023 Pituitary Society guidelines but at odds with a French multi-platform study indicating that this phenomenon is rarely seen with contemporary assays [17]. Potential explanations for this discrepancy between practice and the latest evidence include clinician inertia, differing local experience relative to the French cohort, variable international availability of modern assays, and preferential adherence to consensus guidelines over isolated studies.

The most notable finding in prolactinoma practices amongst endocrinologists and neurosurgeons was that dopamine agonists remained the preferred first-line therapy across a range of microprolactinoma and Knosp grade 1 macroprolactinoma scenarios despite the 2023 Pituitary Society guidelines promoting surgery to an equal first-line therapeutic alternative to dopamine agonists in these settings [10]. Whether this preference changes with increased dissemination of the guidelines remains to be seen. Medical versus surgical preference may be influenced by ongoing investigation of the possibility of cabergoline-induced prolactinoma fibrosis which has been one of the main arguments in favour of upfront surgery for prolactinomas rather than an initial attempt at dopamine agonist therapy [10, 27]. Ongoing investigations may also lead to improved characterisation of the side effect profile of dopamine agonists. Conversely, cabergoline was selected as the preferred therapy by only 53% of participants for giant prolactinomas with visual loss and by only 38% for cystic macroprolactinomas with visual loss. This is again discordant to the Pituitary Society guidelines which advocate for the use of cabergoline in both settings [10], and may change with increasing awareness and adoption of the guidelines. Longitudinal studies of surgical versus medical therapy across a variety of prolactinoma scenarios may help both clinicians and patients to choose the most appropriate treatment modality in each situation.

There was considerable variability in screening for cabergoline-associated valvulopathy, reflecting mixed evidence for this potential toxicity in the prolactinoma setting compared to the Parkinson’s disease setting where it has been a long-established risk due to higher dose exposures [29]. There was also variability in prolactinoma surveillance during pregnancy, reflecting differing international guideline recommendations [10, 13]. Large prospective studies of pregnant women with prolactinomas may help address this discrepancy, acknowledging that prolactinomas are predominantly a disease in reproductive age women. Other areas of variability were compounded by a lack of corresponding guidelines on the topics, including dopamine agonist commencement and weaning regimens and whether to interrupt cabergoline during radiotherapy to promote prolactinoma cell division and hence susceptibility to irradiation versus cabergoline continuation to control tumour volume and hence the irradiation target volume.

Limitations of our study include the potential for response bias with the participant group being more likely to be engaged in prolactinoma care compared to clinicians who did not complete the survey. This is supported by the high case volume of neurosurgeon participants with a median rate of 60 pituitary operations annually, although the prolactinoma caseload of endocrinologist participants was more variable with 57% seeing fewer than 6 prolactinoma cases per month and 42% seeing 6 or more per month. Our multi-pronged recruitment methodology enabled us to reach a large sample size but prevented us from determining how many clinicians chose to not participate in the survey. This lack of a denominator makes it difficult to determine the generalisability of our findings. Participants might have responded with what they believe they should do or be aware of – rather than responding with their actual practice – which might inflate estimates of guideline adherence. We also note that although Question 12 offered response options including cabergoline, surgery and an equal-weighting option, the question wording asked for clinicians’ usual initial management strategy and did not explicitly distinguish this from the option ultimately chosen by patients following shared decision-making; it is possible that some respondents interpreted the question in the latter sense. Furthermore, factors beyond adenoma size and Knosp grade - including fertility intentions, tolerance of side effects and individual patient preference - also influence this decision in practice and were not captured by our case-based question format. Finally, as a cross-sectional survey, the study captures practices and opinions at a single point in time rather than necessarily reflecting stable patterns of clinical care.

Although this survey was conducted approximately 20 months after publication of the Pituitary Society prolactinoma guidelines, dissemination and adoption of new guidance into routine clinical practice typically occurs over a longer timeframe. Our findings therefore most likely reflect practice patterns that largely predate substantial guideline uptake, rather than constituting a definitive test of guideline adherence; we intend to repeat this survey in several years to delineate the trajectory of practice change over time. Progressive guideline adoption may vary between regions and according to clinician pituitary subspecialisation. As the PTCOE model is progressively adopted internationally [12, 30–34], we will be able in future to delineate survey responses according to whether clinicians are in a PTCOE or not. Future iterations of this survey could usefully ask neurosurgeons to report the proportion of their annual pituitary operative volume attributable specifically to prolactinomas, which would better contextualise surgical experience with this tumour subtype. For now, these data show a broad snapshot of prolactinoma practices worldwide.

In conclusion, this survey dataset builds on the Pituitary Society’s recent prolactinoma guidelines, serving as a roadmap for future research and clinical service improvement in prolactinoma care globally. It provides an evidence base to advocate for resources, such as expansion of MDT services to routinely incorporate patients with prolactinomas who have otherwise been a large patient group typically excluded from dedicated pituitary services. Repeated surveys may help delineate such progress.

## Supplementary Information

Below is the link to the electronic supplementary material.

Supplementary Material 1

## Data Availability

No datasets were generated or analysed during the current study.
