# Supplementary material for: Prolactinoma assessment and management: an international multidisciplinary cross-sectional survey of current practice
Source: Pituitary. 2026 Sep 16;29(5):156. doi: 10.1007/s11102-026-01757-8 (PMC13582103; doi:10.1007/s11102-026-01757-8)
Supplement: Supplementary file 1 [file 11102_2026_1757_MOESM1_ESM.pdf]

Prolactinoma Assessment and Management: An International Multidisciplinary Cross-Sectional Survey of Current Practice

SUPPLEMENTAL DATA

Supplemental Table 1. Survey questions, answer options and collated responses

Question Number	Question	Answer Options
1	What is your specialty?	a) Endocrinology 84%<br>b) Neurosurgery 16%
2	Which country do you currently work in?	(free text)
3	What kind of institution do you predominantly work in?	a) Tertiary university hospital 80%<br>b) Other hospital 6%<br>c) Private practice 13%<br>d) Other 2%
4	How many patients with prolactinomas do you see in a typical month?	a) <2 16%<br>b) 2-6 42%<br>c) >6 41%
5	Regarding pituitary MDT meetings:	
5a	There is a regular meeting in my region	a) Yes 92%<br>b) No 2%<br>c) Unsure 6%
5b	I can attend this meeting	a) Yes 81%<br>b) No 13%<br>c) Unsure 6%
5c	I can directly refer prolactinoma patients for MDT discussion	a) Yes 83%<br>b) No 11%<br>c) Unsure 6%
5d	I refer most or all patients with a newly diagnosed prolactinoma for MDT discussion regarding first-line therapy	a) Yes 20%<br>b) No 78%<br>c) Unsure 3%
6	Which aspects are usually included in prolactinoma MRI scans performed in your local radiology practice/s?	
6a	Fine cut imaging through the pituitary	a) Yes 92%<br>b) No 2%<br>c) I don't know 6%
6b	Gadolinium administration	a) Yes 96%<br>b) No 3%<br>c) I don't know 2%
6c	Dynamic sequence	a) Yes 70%<br>b) No 19%<br>c) I don't know 11%
6d	Maximal diameter reported	a) Yes 93%<br>b) No 4%<br>c) I don't know 4%
6e	All 3 diameters reported	a) Yes 62%<br>b) No 32%<br>c) I don't know 6%

6f	Knosp grade reported	a) Yes 39%<br>b) No 55%<br>c) I don't know 6%
6g	Review by a neuroradiologist	a) Yes 67%<br>b) No 25%<br>c) I don't know 9%
7	Which dopamine agonists are usually available to treat prolactinomas in your country? (tick all that apply)	a) Cabergoline 100%<br>b) Quinagolide 23%<br>c) Bromocriptine 66%<br>d) Other 0%
8	Which features are usually reported in your local pituitary histopathology reports?	
8a	Hormone Staining	a) Yes 96%<br>b) No 1%<br>c) I don't know 3%
8b	Transcription Factor Staining	a) Yes 64%<br>b) No 21%<br>c) I don't know 15%
8c	Granulation Pattern	a) Yes 70%<br>b) No 15%<br>c) I don't know 15%
8d	Ki-67 index	a) Yes 88%<br>b) No 7%<br>c) I don't know 4%
8e	MGMT staining	a) Yes 15%<br>b) No 58%<br>c) I don't know 27%
9	What local radiotherapy services are usually available for your patients? (tick all that apply)	a) Conventional fractionated radiotherapy 86%<br>b) Gamma knife stereotactic radiotherapy 71%<br>c) LINAC stereotactic radiosurgery (eg. Cyberknife) 45%<br>d) Proton beam therapy 20%<br>e) None 1%<br>f) I don't know 6%
10	Do your patients usually have access to germline genetic testing if you suspect a heritable pituitary adenoma condition?	a) Yes, I refer patients to a local clinical genetic service who assess patients and arrange genetic testing 54%<br>b) Yes, I arrange genetic testing myself 36%<br>c) No 6%<br>d) I don't know 4%
11	Which additional biochemical tests do you perform to clarify the diagnosis of prolactinoma?	
11a	Repeat prolactin	a) Yes 90%<br>b) No 2%<br>c) Sometimes 8%<br>d) I don't know 1%
11b	Repeat prolactin on another platform	a) Yes 10%<br>b) No 54%<br>c) Sometimes 32%<br>d) I don't know 4%
11c	IGF-1 level	a) Yes 85%

		b) No 5%<br>c) Sometimes 8%<br>d) I don't know 2%
11d	PEG precipitation for macroprolactin	a) Yes 62%<br>b) No 9%<br>c) Sometimes 24%<br>d) I don't know 5%
11e	Serial dilution for hook effect	a) Yes 37%<br>b) No 23%<br>c) Sometimes 38%<br>d) I don't know 2%
11f	Cannulated prolactin test	a) Yes 12%<br>b) No 55%<br>c) Sometimes 18%<br>d) I don't know 15%
12	Which first-line treatment strategy do you usually employ in the following select scenarios?	
12a	Microprolactinoma and hyperprolactinaemia-related amenorrhoea in a premenopausal woman, not wishing to conceive, no galactorrhoea	a) Cabergoline 75%<br>b) Surgery 0%<br>c) Equal between surgery and cabergoline 8%<br>d) Sex hormone replacement (oral contraceptives) 10%<br>e) I don't treat such tumours 4%<br>f) Bromocriptine 1%
12b	Microprolactinoma and hyperprolactinaemia-related amenorrhoea in a premenopausal woman, wishing to conceive	a) Cabergoline 74%<br>b) Surgery 4%<br>c) Equal between surgery and cabergoline 14%<br>d) Sex hormone replacement (oral contraceptives) 0%<br>e) I don't treat such tumours 3%<br>f) Bromocriptine 6%
12c	Microprolactinoma and hyperprolactinaemia-related hypogonadism and galactorrhoea	a) Cabergoline 82%<br>b) Surgery 2%<br>c) Equal between surgery and cabergoline 13%<br>d) Sex hormone replacement (oral contraceptives) 1%<br>e) I don't treat such tumours 2%<br>f) Bromocriptine 1%
12d	Macroprolactinoma with Knosp grade 1 cavernous sinus invasion	a) Cabergoline 76%<br>b) Surgery 5%<br>c) Equal between surgery and cabergoline 17%<br>d) Sex hormone replacement (oral contraceptives) 0%<br>e) I don't treat such tumours 2%<br>f) Bromocriptine 0%
12e	Macroprolactinoma with Knosp grade 2 cavernous sinus invasion	a) Cabergoline 84%<br>b) Surgery 4%<br>c) Equal between surgery and cabergoline 10%<br>d) Sex hormone replacement (oral contraceptives) 0%<br>e) I don't treat such tumours 2%<br>f) Bromocriptine 0%

12f	Macroprolactinoma with Knosp grade 3-4 cavernous sinus invasion	a) Cabergoline 83%<br>b) Surgery 6%<br>c) Equal between surgery and cabergoline 9%<br>d) Sex hormone replacement (oral contraceptives) 0%<br>e) I don't treat such tumours 2%<br>f) Bromocriptine 0%
12g	Giant prolactinoma with intact visual fields	a) Cabergoline 85%<br>b) Surgery 6%<br>c) Equal between surgery and cabergoline 7%<br>d) Sex hormone replacement (oral contraceptives) 0%<br>e) I don't treat such tumours 1%<br>f) Bromocriptine 1%
12h	Giant prolactinoma with bitemporal hemianopia	a) Cabergoline 53%<br>b) Surgery 27%<br>c) Equal between surgery and cabergoline 17%<br>d) Sex hormone replacement (oral contraceptives) 1%<br>e) I don't treat such tumours 3%<br>f) Bromocriptine 0%
12i	Cystic macroprolactinoma with intact visual fields	a) Cabergoline 78%<br>b) Surgery 6%<br>c) Equal between surgery and cabergoline 12%<br>d) Sex hormone replacement (oral contraceptives) 0%<br>e) I don't treat such tumours 2%<br>f) Bromocriptine 1%
12j	Cystic macroprolactinoma with bitemporal hemianopia	a) Cabergoline 38%<br>b) Surgery 39%<br>c) Equal between surgery and cabergoline 19%<br>d) Sex hormone replacement (oral contraceptives) 1%<br>e) I don't treat such tumours 3%<br>f) Bromocriptine 0%
13	The final section is specialty specific. Please indicate your specialty of practice:	a) Endocrinology 84%<br>b) Neurosurgery 16%
14	In the presence of ongoing visible tumour, what PRL levels do you aim for during dopamine agonist treatment?	a) Suppressed PRL 42 20%<br>b) PRL in the reference range 79%<br>c) I'm not sure 2 1%
15	What is your typical starting dose of cabergoline?	a) 0.25mg weekly 20%<br>b) 0.25mg twice weekly 44%<br>c) 0.5mg weekly 24%<br>d) 0.5mg twice weekly 7%<br>e) Other 4%
16	When prescribing dopamine agonists, which potential consequences do you routinely discuss with patients before starting therapy?	
16a	GI Side Effects	a) Yes 81%<br>b) No 13%

		c) Sometimes 6%
16b	Vasodilatory Side Effects	a) Yes 68%<br>b) No 24%<br>c) Sometimes 8%
16c	Mood Disturbance	a) Yes 62%<br>b) No 22%<br>c) Sometimes 16%
16d	Impulse Control Disorder	a) Yes 70%<br>b) No 16%<br>c) Sometimes 14%
16e	Cardiac Valvulopathy	a) Yes 56%<br>b) No 21%<br>c) Sometimes 24%
16e	CSF Rhinorrhoea	a) Yes 23%<br>b) No 39%<br>c) Sometimes 28%
16f	Return of fertility	a) Yes 92%<br>b) No 2%<br>c) Sometimes 6%
17	Regarding the risk of cabergoline-associated valvulopathy, which of the following best represents your approach at ***baseline*** (before or soon after commencing therapy)?	a) Baseline echocardiography only if abnormalities detected on examination 23%<br>b) Baseline echocardiography if abnormalities detected on examination or anticipated to have prolonged high-dose cabergoline therapy (>2mg weekly) 42%<br>c) Baseline echocardiography in all 15%<br>d) I do not perform a baseline cardiovascular exam or echocardiography 20%<br>e) I'm not sure 0%
18	Regarding the risk of cabergoline-associated valvulopathy, which of the following approaches do you take on ***follow-up***? (tick all that apply)	a) Annual cardiac examination 22%<br>b) Echocardiography if new heart murmur 37%<br>c) Echocardiography every 2-3 years if weekly dose >2mg 62%<br>d) Echocardiography every 5-6 years if weekly dose 1-2mg 24%<br>e) Echocardiography every 5-6 years if weekly dose >2mg 11%<br>f) I do not routinely perform a cardiac examination 17%<br>g) I'm not sure 1%
19	If a woman becomes pregnant whilst taking cabergoline for a microprolactinoma, what is your approach? (tick all that apply)	a) Cease dopamine agonist therapy 97%<br>b) Continue cabergoline 2%<br>c) Switch to bromocriptine 2%<br>d) Clinically assess halfway through pregnancy 10%<br>e) Clinically assess in each trimester 47%<br>f) Perform visual field perimetry halfway through pregnancy 11%<br>g) Perform visual field perimetry in each trimester 17%<br>h)I'm not sure 1%
20	If a woman becomes pregnant whilst taking cabergoline for a macroprolactinoma currently in	a) Cease dopamine agonist therapy and monitor closely instead 33%

	contact with the optic chiasm, what is your approach?	b) Switch to bromocriptine at an equivalent dose 18%<br>c) Continue cabergoline 42%<br>d) I'm not sure 7%
21	In which of the following scenarios do you offer the option of being off dopamine agonist therapy to permit breastfeeding?	a) Previous prolactinoma with no evidence of tumour on the last scan 93%<br>b) Microprolactinoma 95%<br>c) Macroprolactinoma without suprasellar extension 65%<br>d) Macroprolactinoma in contact but not compressing the optic chiasm 23%<br>e) Macroprolactinoma compressing the optic chiasm 2%<br>f) None of the above (I always recommend dopamine agonist continuation) 2%<br>g) I'm not sure 4%
22	In which of the following scenarios, do you consider dopamine agonist withdrawal?	
22a	Microprolactinoma, 2 years of dopamine agonist, normal serum PRL, partial tumour shrinkage	a) Yes 39%<br>b) No 56%<br>c) Unsure 5%
22b	Microprolactinoma, 2 years of dopamine agonist, normal serum PRL, complete tumour resolution	a) Yes 93%<br>b) No 5%<br>c) Unsure 1%
22c	Macroprolactinoma, 2 years of dopamine agonist, normal serum PRL, partial tumour shrinkage	a) Yes 8%<br>b) No 88%<br>c) Unsure 4%
22d	Macroprolactinoma, 2 years of dopamine agonist, normal serum PRL, complete tumour resolution	a) Yes 63%<br>b) No 31%<br>c) Unsure 7%
23	If you decide to withdraw cabergoline therapy, do you gradually wean the dose before cessation?	a) No 21%<br>b) Yes, down to 0.5mg weekly then stop 14%<br>c) Yes, down to 0.25mg weekly then stop 52%<br>d) Yes, down to 0.25mg monthly then stop 13%
24	In which of the following dopamine agonist-induced scenarios, do you discuss the option of switching to surgical management (assuming prolactinoma treatment is required and the tumour is resectable)?	
24a	Fatigue	a) Yes 8%<br>b) No 63%<br>c) Sometimes 30%
24b	Nausea and/or anorexia	a) Yes 32%<br>b) No 29%<br>c) Sometimes 39%
24c	Postural lightheadedness	a) Yes 23%<br>b) No 36%<br>c) Sometimes 41%
24d	Depression	a) Yes 39%<br>b) No 29%<br>c) Sometimes 22%

24e	Impulse control disorders e.g., hypersexuality, binge eating, pathological gambling	a) Yes 78%<br>b) No 7%<br>c) Sometimes 16%
24f	Persistent but stable hyperprolactinaemia with a tumour remnant away from the optic chiasm after 2 years of maximally tolerated dopamine agonist therapy	a) Yes 34%<br>b) No 35%<br>c) Sometimes 31%
24g	Growing tumour remnant and/or rising prolactin after 2 years of maximally tolerated dopamine agonist therapy	a) Yes 89%<br>b) No 3%<br>c) Sometimes 8%
25	Do you have access to a local neurosurgeon with subspecialty training (such as a fellowship) in pituitary surgery to whom you can refer your patients?	a) Yes 95%<br>b) No 3%<br>c) Unsure 2%
26	If a patient has a postoperative prolactinoma remnant that is being treated with cabergoline and now requires radiotherapy, how do you manage cabergoline use around the time of radiotherapy?	a) Stop cabergoline 1 week before commencing radiotherapy 8%<br>b) Stop cabergoline 6 weeks before commencing radiotherapy 17%<br>c) Continue cabergoline throughout 51%<br>d) Switch to bromocriptine 0%<br>e) I don't treat such cases 24%
27	Optional: If you would like to be acknowledged in publications of this study, please list your name and primary institution below. Your details will be handled separately to your responses so that your responses will remain anonymous	(free text)
28	I confirm that I have answered all questions truthfully and consent to use of my anonymous responses in future publications. I will not repeat the survey.	a) Yes 100%
29	Have you undertaken subspecialty training (such as a fellowship) in pituitary surgery?	a) Yes 82%<br>b) No 18%
30	How many pituitary operations do you perform in a typical year?	(free text)
31	How do you typically undertake resection of pituitary tumours?	a) Endoscopic transsphenoidal technique 85%<br>b) Microscopic transsphenoidal technique 10%<br>c) Either 5%
32	Describe scenarios where you would use a microscopic approach	(free text)
33	Describe scenarios where you would use an endoscopic approach	(free text)
34	What are the typical surgery wait times in the following scenarios?	
34a	Functioning microadenoma	a) <1 week 3%

		b) 1-4 weeks 39%<br>c) >4 weeks 51%<br>d) >3 months 8%
34b	Pituitary macroadenoma with visual loss	a) <1 week 28%<br>b) 1-4 weeks 62%<br>c) >4 weeks 10%<br>d) >3 months 0%
34c	CSF leak repair	a) <1 week 90%<br>b) 1-4 weeks 8%<br>c) >4 weeks 3%<br>d) >3 months 0%
35	What do you feel is the most common surgical indication for prolactinomas in your personal practice?	a) Patient preference 23%<br>b) Dopamine agonist intolerance 49%<br>c) Dopamine agonist resistance 18%<br>d) Other 10%
36	How often do you find that tumour resection is hampered by apparent dopamine agonist-associated tumour fibrosis?	
36a	Bromocriptine	a) Almost always 3%<br>b) Frequently 8%<br>c) Occasionally 23%<br>d) Almost never 18%<br>e) Uncertain 49%
36b	Cabergoline	a) Almost always 3%<br>b) Frequently 15%<br>c) Occasionally 41%<br>d) Almost never 26%<br>e) Uncertain 15%
37	Optional: If you would like to be acknowledged in publications of this study, please list your name and primary institution below. Your details will be handled separately to your responses so that your responses will remain anonymous	(free text)
38	I confirm that I have answered all questions truthfully and consent to use of my anonymous responses in future publications. I will not repeat the survey.	a) Yes 100%

Supplemental Table 2. Acknowledgement of named survey participants

Adriana Ioachimescu
Alberto M Pereira
Alistair Jukes
Almudena Vicente
Ana Melgarejo
Anastasia Athanasoulia-Kaspar
Andreu Simó Servat
Andrew S Powlson
Anna Aulinas
Annika Mascarenhas
Anthony Roberts
Aparna Pal
Ashley Grossman
B L Herrmann
Bahar Force
Bernard Corenblum
Betina Biagetti
Brian Fredericks
Brindha Shivalingam
Camilla Schalin-Jäntti
Carmela Caputo
Carmen Tenorio-Jiménez
Caroline Jung
Cesar L Boguszewski
Charlotte Höybye
Christof Kloos
Christopher Rowe
Cristina Lamas
Dominique Maiter
Edy Kornelius
Elena Vorona
Esra Hatipoglu
Eva Venegas Moreno
Federico Gatto
Felicia Hanzu
G Edward Vates
G Vlotides
Garni Barkhoudarian
Gemma Xifra
Georgia Ntali
Gerald Raverot
Gudrun Caspar-Bell
Heidi Immonen
Henrik Falhammar
Hidenori Fukuoka
Hiroshi Nishioka
Ignacio Bernabéu
Iina Yliaska
Ilan Shimon
Isabella Nasi-Kordhishti
Ismat Shafiq
James King
Javier Aller

Joanna Spencer-Segal
Joao Paulo Almeida
Jochen Schopohl
John Ayuk
Juan C Fernández-Miranda
Julia Zuarth Vazquez
Katharina Schilbach
Kylie McLachlan
Laurent Vroonen
Leonidas Duntas
Linus Haberbosch
Lourdes Garcia
Lucas Carlstrom
Lukas van Baal
Luma Ghalib
M Dolores Ollero
Manel Puig-Domingo
Marco Losa
María Calatayud Gutiérrez
Maria Chiara Zatelli
Maria Fleseriu
Mark McLean
Marta Araujo-Castro
Martin Fassnacht
Matthias Hepprich
Michio Otsuki
Miguel Paja
Minna Koivikko
Minna Soinio
Minna Takkunen
Mirtha A Guitelman
Montserrat Marques-Pamies
Mussa Hussain Almalki
N Biermasz
N Thomas
Nesrin Uksul
Ni Ni Khin
Nick Phillips
Orsalia Alexopoulou
Oskar Ragnarsson
P L Chan
Per Dahlqvist
Peter Mews
Peter Shane Hamblin
Pia Burman
Pietro Maffei
Pretissha Harrichund
Ramesh Nair
Reeta Rintamäki
Richard Carroll
Roman Rotermund
Ruth Ronneberger
Sandi Powell
Santosh Poonnoose
Sari Mäkimattila

Scott Baker
Shaan Raza
Shingo Fujio
Shozo Yamada
Simon Cudlip
Sinan Barazi
Stephen T Magill
Susan Webb
Theofanis Giannis
Thierry Brue
Thomas L Beaumont
Timo Deutschbein
Tom Wilkinson
Ursula B Kaiser
Victor Hantelius
Victoria Alcázar Lázaro
Warrick Inder
Wolfram Karges
Yeung-Ae Park
Yona Greenman
Yutaka Takahashi
